# Microbial lipid fermentation of *Trichosporon cutaneum* in high saline water

**DOI:** 10.1186/s40643-021-00424-z

**Published:** 2021-08-10

**Authors:** Luhuan Sun, Shuai Shao, Jie Bao

**Affiliations:** State Key Laboratory of Bioreactor Engineering, East China University of Science and Technology, 130 Meilong Road, Shanghai, 200237 China

**Keywords:** *Trichosporon cutaneum*, Microbial lipid, Saline water, Phenol degradation, Cell separation

## Abstract

Fermentative production of microbial lipid requires high fresh water input. The utilization of high saline seawater or industrial wastewater is an important alternative to reduce the freshwater consumption. This study revealed that oleaginous yeast *Trichosporon cutaneum* was tolerant to a high salinity up to 130 g/L of NaCl after long-term adaptive evolution. Lipid fermentation of *T. cutaneum* in seawater achieved the lipid production of 31.7 g/L with approximately 36% greater than that in freshwater. The saline water containing phenol was also tested for lipid fermentation and 23.6 g/L of lipid was produced simultaneously with the complete biodegradation of phenol. An interesting phenomenon was also observed that the yeast cells spontaneously segregated onto the upper surface of the saline water. This study extended the lipid fermentation options with practical application potentials.

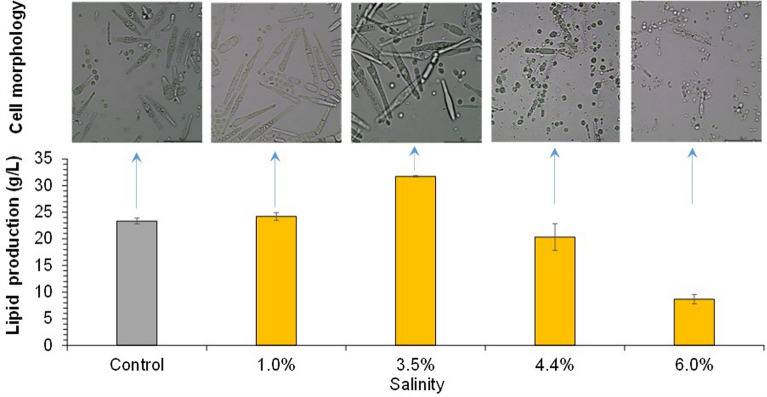

## Introduction

Microbial lipid provides an important alternative of vegetable lipid feedstock for production of aviation fuel and biodiesel (Ju et al. 2016; Li et al. 2008). Oleaginous yeast is the major cell factory for fermentative production of microbial lipid. However, microbial lipid fermentation requires high fresh water input then generates large amount of wastewater, resulting in the heavy burdens of fresh water usage and downstream wastewater treatment (Yen et al. 2016).

One practical solution is to use saline water such as seawater or industrial saline wastewater as alternative of fresh water. Seawater has the typical salinity of 3.5% and has been used for lipid production by microalgae (Sabeela Beevi and Sukumaran, 2015; Takagi et al. 2006). Oleaginous yeasts *Yarrowia lipolytica* (Dobrowolski et al. 2019) and *Rhodotorula glutinis* (Yen et al. 2016) were also tested in seawater, but their cell growth was significantly suppressed. Wastewater from textiles, pharmaceuticals, tannery, petroleum, petrochemical and pickled vegetable industries generally has a wide range of salinity from 0.2 to 15% (Ng et al. 2015; von Alvensleben et al. 2013; Yurtsever et al. 2016; Lefebvre and Moletta 2006; Kubo et al. 2001) and contains organic impurities such as phenol (Ren et al. 2018). High saline tolerance and toxin biodegradability of oleaginous yeasts is the pre-condition of saline water used for microbial lipid production.

This study investigated the use of seawater and phenol-containing saline water for lipid fermentation by a robust oleaginous yeast *Trichosporon cutaneum* (Hu et al. 2018; Wang et al. 2016). *T. cutaneum* was found to be tolerant to very high salinity after the long-term adaptive evolution. Adaptive evolution provides a practical method to elevate the robustness of microorganisms under tolerance or inhibitions. For oleaginous yeast strains, adaptive evolution is also a feasible way for improving the lipid accumulation capacity under specific stress such as salinity (Daskalaki et al. 2019). Lipid fermentation of *T. cutaneum* was conducted under typical salinities of seawater and phenol-containing saline water. An interesting phenomenon was found that the yeast cells floated on upper layer of fermentation liquid in high salt conditions. The result provided a practical and cost-effective method for microbial lipid production using saline water.

## Materials and methods

### Water sources and reagents

Seawater was taken from East China Sea (30.819° N, 121.528° E) at Fengxian Beach, Shanghai, China. The salinity of seawater was 0.98% and the main metal ions included 3.2 g/L of Na^+^, 0.43 g/L of Mg^2+^, 0.17 g/L of Ca^2+^, 0.11 g/L of K^+^. The seawater was adjusted to different salinities by adding NaCl. The phenol-containing saline water was prepared by adding 35 g/L NaCl and the given amount of phenol into freshwater.

Peptone and yeast extract were purchased from Oxoid Co. (Hampshire, UK). Phenol and other analytical grade chemicals were purchased from Shanghai Titan Scientific Co. (Shanghai, China).

### Strains, media and culture conditions

*Trichosporon cutaneum* ACCC 20271 was obtained from Agricultural Culture Collection of China (ACCC, http://www.accc.org.cn), Beijing, China. *T. cutaneum* MP11 was a mutant strain obtained in our lab and stored in China General Microorganisms Collection Center (CGMCC, http://www.cgmcc.net), Beijing, China, with the registration number of 20481.

YPD medium and synthetic medium referred to Hu et al. (2018), but 60 g/L of glucose was added to the synthetic medium instead of inhibitor. The fermentation medium in 3-L bioreactor was supplemented with 150 g/L of glucose, and the remaining components were all added twice as much as the synthetic medium.

### Lipid fermentation and extraction

Lipid fermentation was carried out in a 3-L bioreactor (Baoxing Biotech, Shanghai, China) with a working volume of 800 mL. The fermentation was maintained for 120 h at 30 ℃ and 450 rpm with Rushton impeller and pH 5.0 by using 5 M NaOH and 4 M HCl solutions.

The microbial lipid was extracted by the methanol–chloroform method (Wang et al. 2016).

### Analytical methods

Glucose was measured using the biosensor analyzer SBA-40D (Shandong Academy of Sciences, Jinan, China). The metal ions were measured by ICP-OES (Agilent, California, USA) (Fingerova and Koplik 1999). Cell growth was detected by the method depicted in Jin et al. (2019). Phenol was analyzed using HPLC (Shimadzu, Kyoto, Japan) according to the method described in Kilic (2009).

## Results and discussion

### Saline tolerance evolution of *T. cutaneum* under different salinities

**Fig. 1:**
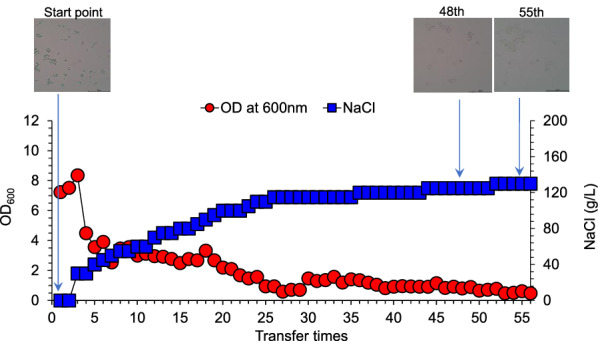
Adaptive evolution of *T. cutaneum* ACCC 20271 under varying saline conditions. Synthetic medium supplemented with increasing sodium chloride (from 0 to 130 g/L). The transfer was conducted every 72 h at 30 ℃ into fresh synthetic medium at 10% (v/v) inoculation. Cell morphology was observed with an optical electron microscope (×100)

Saline tolerance of *T. cutaneum* ACCC 20271 was examined in the 168-day long-term adaptive evolution (Fig. 1). The salinity was gradually increased by adding sodium chloride into synthetic medium. The results show that *T. cutaneum* tolerated up to 130 g/L of NaCl, an extremely high salinity. The advantage of adaptive evolution is mainly the cell growth in high saline condition. This experiment mainly focused on the evaluation of saline tolerance in the long-term adaptive evolution. In the transfer time 25, the salt concentration in the medium reached 115 g/L; in the transfer time 55, the salt concentration reached 130 g/L. The cell growth maintained constant although the salt concentration increased 13% in this period. The cell growth decreased with the increasing NaCl concentration, but still in the relatively normal growth period. The cell morphology maintained relatively unchanged when NaCl was below 125 g/L, till the cell corruption at 130 g/L of NaCl. *T. cutaneum* is an environmental microorganism and has strong adaptability to various conditions. This study conducted a preliminary evaluation on lipid fermentation of *T. cutaneum* under high saline tolerance. The high salinity tolerance of *T. cutaneum* is speculated to come from the capacity of *T. cutaneum* cells of strong Na^+^/H^+^ anti-transport activity to pump the intracellular Na^+^ into the extracellular medium under high saline condition. The molecular biology mechanism is under investigation and expected to be available in the near future.

**Fig. 2:**
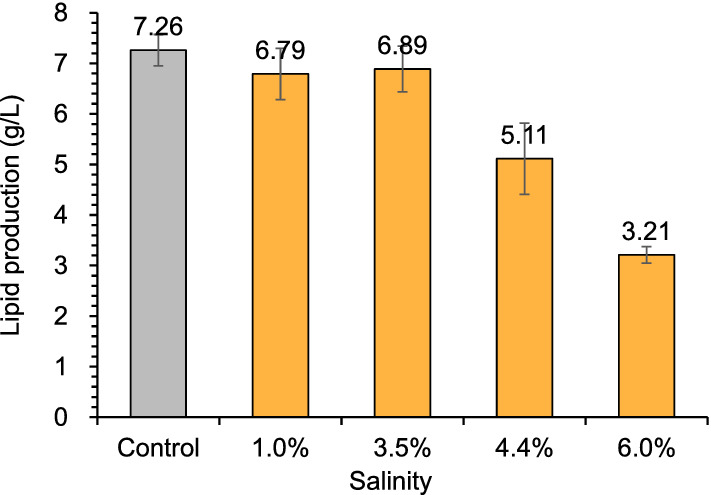
Lipid production of *T. cutaneum* ACCC 20271 at different salinities. The lipid production was measured after 120 h in a 3-L bioreactor with a work volume of 800 mL, 10% (v/v) inoculum size, 30 °C, 450 rpm, pH 5.0 by 5 M NaOH and 4 M HCl, and the aeration at 1.0 vvm. Fresh water was used as the control. All experiments were performed in duplicate

Then *T. cutaneum* ACCC 20271 was used for lipid fermentation in 3-L bioreactor under different salinities. These salinities ranged from 1.0 to 6.0%, corresponding to the salinities of textiles and electro-dialysis wastewater (1.0%) (Tiwari et al. 2003; Vo et al. 2020; Maeng et al. 2018), aquaculture and seawater (3.5%) (Zaky et al. 2018; Ren et al. 2017; Song et al. 2018), seawater in arid areas and petrochemical (4.4%) (Jorfi et al. 2019; Tiwari et al. 2003), tannery and pharmaceutical factories (6.0%) (Lefebvre et al. 2005; Ng et al. 2015), respectively. Figure 2 shows that the lipid yields at the lower salinity of 1.0% and 3.5% were similar to that of freshwater, then decreased when the increasing salinity of 4.4% and 6.0%. The results indicate that *T. cutaneum* well tolerated the salinity of 3.5%, but 6.0% salinity obviously inhibited its lipid production.

### Lipid fermentation of *T. cutaneum* under different salinities

**Fig. 3:**
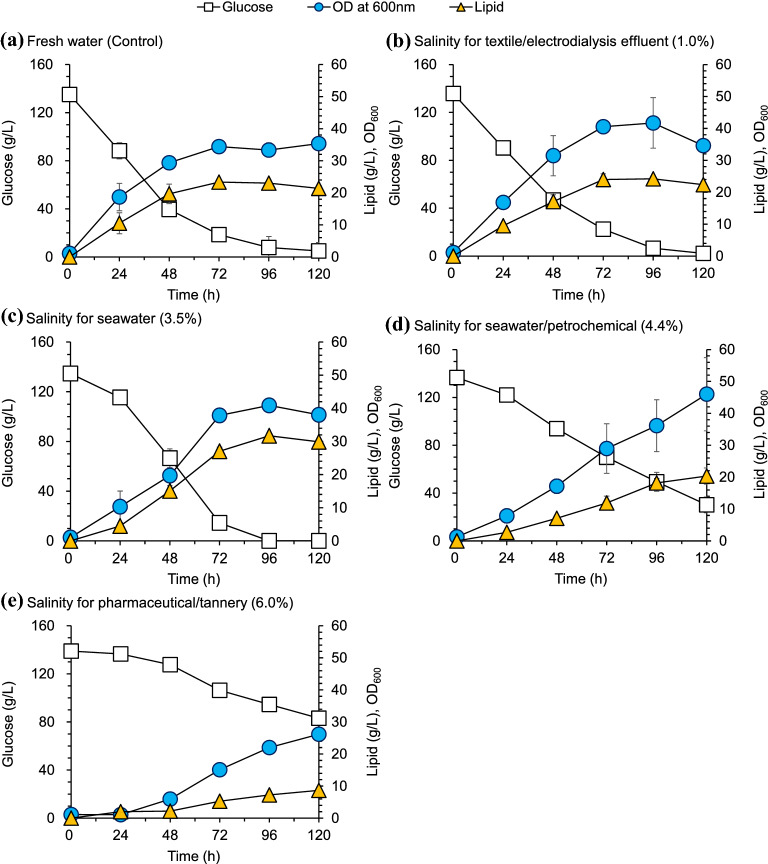
Cell growth, glucose and lipid production of *T. cutaneum* MP11 under different salinities. Conditions: **a** fresh water, **b** 1.0% salinity, **c** 3.5% salinity, **d** 4.4% salinity, **e** 6.0% salinity, other fermentation conditions were the same as *T. cutaneum*. ACCC 20271. All experiments were performed in duplicate

*T. cutaneum* ACCC20271 has relatively high saline tolerance, but its low lipid production was not suitable for practical lipid fermentation. *T. cutaneum* MP11 was obtained by long-term adaptive evolution and ultra-centrifugation screening with higher lipid accumulation. Therefore, *T. cutaneum* MP11 was used for lipid fermentation under high salinity condition to evaluate its potentials (Fig. 3). The results show that the higher lipid production was observed at 1.0% and 3.5% salinity. With further increase of salinity (up to 4.4% and 6.0%), both the glucose consumption rate and the lipid accumulation decreased. The maximum lipid production (31.7 g/L) was obtained at 3.5% salinity, which was even 36% greater than that using fresh water (23.3 g/L). Only few studies were reported on the microbial lipid fermentation in high saline water. Yen et al. (2016) studied that the growth of *R. mucilaginosa* in seawater using crude glycerol and the lipid production reached 12.2 g/L. The lipid production (31.7 g/L) is the highest that has been reported under high saline condition.

**Fig. 4:**
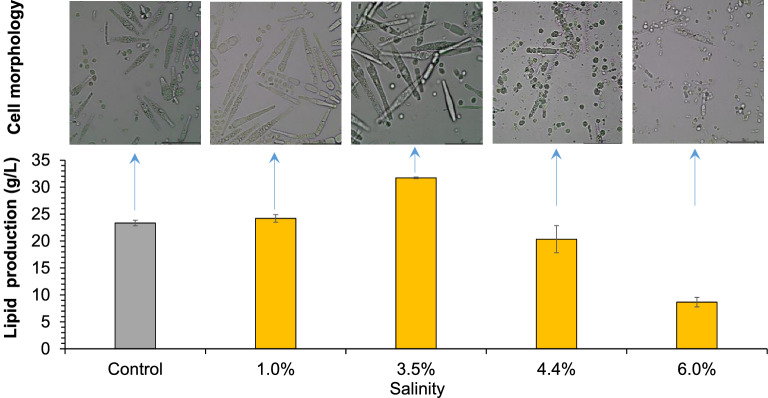
Lipid production and cell morphology of *T. cutaneum* MP11 under different salinities. Lipid production was obtained at 96 h for the control, 1.0% and 3.5% salinity, 120 h for 4.4% and 6.0% salinity. Cell morphology was photographed after 96 h with enlargement of ×100

The cell morphology of *T. cutaneum* MP11 was correlated with the varying salinities (Fig. 4). The yeast cells appeared as long and large rods when the salinity was below 3.5%, then the cells changed to small round balls at the salinity of 4.4%, and finally shrank and died due to a stronger saline osmotic pressure when the salinity reached 6.0%. The results show that high saline conditions induced strong stress on the cell morphology and then changed the lipid accumulation performance.

**Fig. 5:**
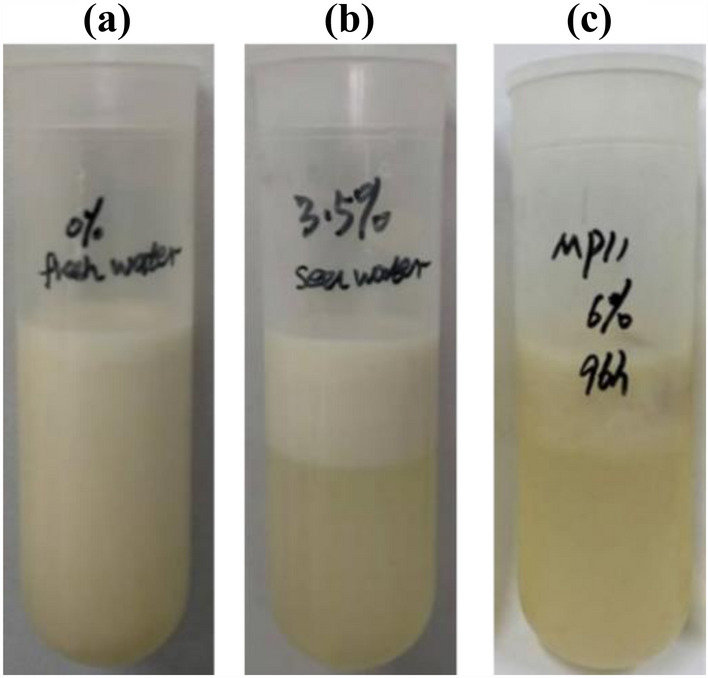
Broth samples of *T. cutaneum* MP11 under different salinities. These samples were cultured for 96 h in **a** fresh water, **b** 3.5% salinity, and **c** 6.0% salinity, respectively

An interesting phenomenon was observed that the *T. cutaneum* cells spontaneously floated on the upper layer of the fermentation broth at high saline conditions, while this phenomenon was not observed in the freshwater medium (Fig. 5). The possible reasons might be the higher lipid content in cells in the saline water with higher density. This phenomenon is important for the recovery of microbial lipid.

### Lipid fermentation of *T. cutaneum* under phenol-containing saline water

**Fig. 6:**
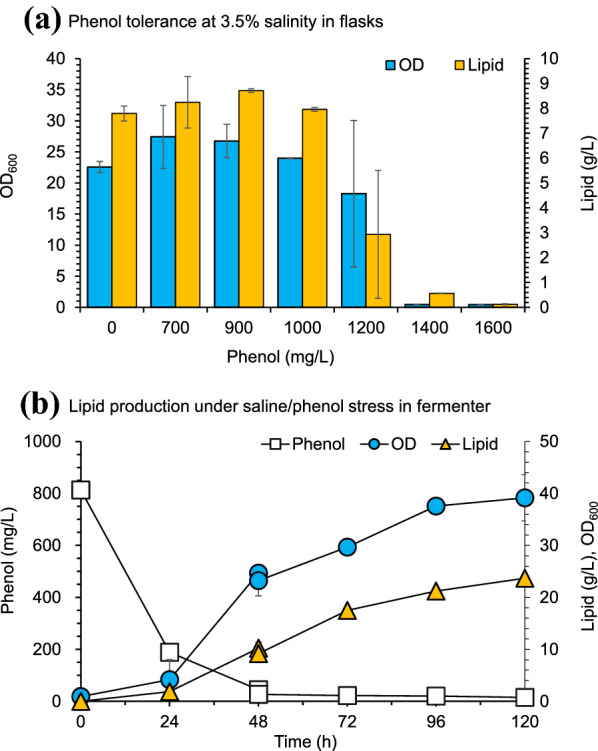
Phenol tolerance and lipid production of *T. cutaneum* MP11 in saline water. **a** Phenol tolerance at 3.5% salinity in flasks. 35 g/L NaCl and defined phenol were added into the synthetic medium. *T. cutaneum* MP11 was cultured at 30 ℃ for 120 h. **b** Lipid production in the medium containing 35 g/L of NaCl and 1000 mg/L of phenol. The initial concentration of glucose is 60 g/L, and added to 150 g/L at 48 h. All experiments were performed in duplicate

Industrial wastewater is the typical saline water containing heavy metals, aromatics and other organic compounds. Phenol is one of the commonly existing organic compounds in various industrial wastewater sources (Jiang et al. 2016; Kamali et al. 2019). The phenol tolerance of *T. cutaneum* MP11 was tested by inoculating into the 3.5% saline water with different initial phenol concentrations ranging from 700 to 1600 mg/L (Fig. 6a). The results show that *T. cutaneum* MP11 was tolerant to 1000 mg/L of phenol. Metabolic pathway of phenol degradation by *T. cutaneum* has been not yet well established in the previous studies. However, the pathway of similar phenolic compounds of *p*-hydroxybenzaldehyde, 4-hydroxy-3-methoxybenzaldehyde (vanillin) and syringaldehyde by *T. cutaneum* has been investigated in our previous studies (Wang et al. 2016; Hu et al. 2018). It is speculated that the phenol degradation by *T. cutaneum* is conducted in a similar way with above phenolic aldehydes. First, phenol is converted to its corresponding alcohol, then further oxidizes into the corresponding acid, and finally to acetyl-CoA or succinyl-CoA as the precursors of TCA cycle or lipid synthesis.

The lipid fermentation of *T. cutaneum* MP11 was carried out in a 3-L bioreactor under the initial phenol concentration of 1000 mg/L (Fig. 6b). The initial glucose was adjusted to 60 g/L, and then added to 150 g/L during the fermentation. The cell growth and lipid production (23.6 g/L) of *T. cutaneum* MP11 were similar to that in freshwater without phenol. Approximately 76.8% of phenol was degraded by *T. cutaneum* MP11 at 24 h and finally approximately consuming all of the phenol added. The result indicates that *T. cutaneum* MP11 not only achieved a high lipid production, but also performed a high phenol degradation under saline wastewater.

## Conclusions

High saline tolerance (130 g/L NaCl) of *T. cutaneum* was found after the long-term adaptive evolution. A higher lipid production of *T. cutaneum* was obtained at 3.5% salinity compared with fresh water and other typical salinities. Moreover, *T. cutaneum* MP11 has the ability of high lipid production (23.6 g/L) and phenol degradation (800 mg/L) under saline wastewater containing phenol. An interesting phenomenon was found that the yeast cells floated on upper layer of fermentation liquid in high salt conditions. The results show that *T. cutaneum* has the potential of lipid production using high saline water.

## Data Availability

All data generated or analyzed during this study are included in this published article.
